# The Oxidative Function of Diferric Transferrin

**DOI:** 10.1155/2012/592806

**Published:** 2012-02-09

**Authors:** Frederick L. Crane, Hans Löw

**Affiliations:** ^1^Department of Biological Science, Purdue University, West Lafayette, IN 47907, USA; ^2^Department of Molecular Medicine and Surgery, Karolinska Institute, 17177 Stockholm, Sweden

## Abstract

There is evidence for an unexpected role of diferric transferrin as a terminal oxidase for the transplasma membrane oxidation of cytosolic NADH. In the original studies which showed the reduction of iron in transferrin by the plasma membranes NADH oxidase, the possible role of the reduction on iron uptake was emphasized. The rapid reoxidation of transferrin iron under aerobic conditions precludes a role for surface reduction at neutral pH for release of iron for uptake at the plasma membrane. The stimulation of cytosolic NADH oxidation by diferric transferrin indicates that the transferrin can act as a terminal oxidase for the transplasma membrane NADH oxidase or can bind to a site which activates the oxidase. Since plasma membrane NADH oxidases clearly play a role in cell signaling, the relation of ferric transferrin stimulation of NADH oxidase to cell control should be considered, especially in relation to the growth promotion by transferrin not related to iron uptake. The oxidase can also contribute to control of cytosolic NAD concentration, and thereby can activate sirtuins for control of ageing and growth.

## 1. Introduction

The remarkable diversity of oxidative activity at the plasma membrane has only recently been brought into focus. Early study of peroxide generation in cells emphasized the role of mitochondria in relation to free radical generation [1, 2]. Other sources of H_2_O_2_ such as xanthine oxidase [3], monoamine oxidase [1], or disulfide bond formations [4] have been recognized, but attributed a lesser role. In the plasma membrane the controlled generation of H_2_O_2_ by leucocytes to fight infection is well established [5, 6]. More recently, the H_2_O_2_ generating capacity in plasma membrane of other types of cells has been recognized and proposed to be the basis for hormone and growth factor response, oxygen sensing [7], and microbicidal action [8–12]. These H_2_O_2_ generating systems have all been related to the NADPH oxidase GP91 phox Gp22 phox enzymes first recognized in neutrophils [5, 6]. Seven variations of this NADPH oxidase have been found and are designated as NOX1-5 and Duo NOX1-2 [9, 13]. The reaction with oxygen in all is catalyzed by the oxidation of low-potential cytochrome b 558. The members of the NOX groups are individually expressed in different tissues [9, 12]. Plasma membranes also have NADH oxidase activity [14–16]. Two forms of NADH oxidase have been extracted from plasma membranes and designated cNOX and tNOX [17]. These oxidases do not use cytochrome b558 as a terminal link to oxygen. The isolated enzyme also acts as a ubiquinol oxidase [18, 19] which may represent its physiological function in the plasma membrane. It could oxidize ubiquinol in the plasma membrane formed by reduction of ubiquinone from cytosolic NADH through NADH cytochrome b_5_ reductase [20, 21]. The activation of the NADH oxidase by diferric transferrin and the relation to cellular control are the subjects of this paper.

## 2. Plasma Membrane Redox Systems

Plasma membranes isolated from rat liver or adipocytes have NADH oxygen oxidoreductase activity (NADH oxidase) [14, 21]. This oxidase activity is not inhibited by agents which inhibit NADH oxidase activity in mitochondria [15, 22] and is stimulated by diferric transferrin [23]. How this oxidase activity is related to other redox activity in plasma membrane remains to be established.

In addition to stimulation by diferric transferrin, the NADH oxidase of rat liver plasma membrane is stimulated by insulin and epidermal growth factor (EGF) [15]. Both the endogenous NADH oxidase and the diferric transferrin-stimulated NADH oxidase are stimulated by coenzyme Q_10_ and are inhibited by coenzyme Q analogs such as piericidin A [15, 21]. Thus, the diferric transferrin stimulated oxidase may require the reduced coenzyme Q oxidase (tNOX) [18, 24] and one of the known NADH dehydrogenases in the plasma membrane for the complete electron transfer chain from NADH to oxygen [19, 20, 25]. Diferric transferrin would be a terminal link to oxygen or can act to increase the reaction with oxygen. There are two NADH dehydrogenases which have been recognized in the plasma membrane. These are (A) the NADH cytochrome b_5_ reductase [20] and (B) the NQ 0-1 (DT diaphorase) [26] which can oxidize both NADH and NADPH. Both of these NADH dehydrogenases can reduce coenzyme Q in the plasma membrane. The occurrence of these dehydrogenases may vary in different plasma membranes [20, 27]. In membranes where either one is present, they would be expected to function in any NADH oxidase system using coenzyme Q and reduced coenzyme Q oxidase to transfer electrons across the membrane. The transfer of electrons to oxygen in the unsupplemented plasma membrane can best be based on the activity of the reduced coenzyme Q oxidase [18]. At this time, the mechanism of reaction and the prosthetic groups involved are not known. There is also evidence for an iron site on the outer surface of the plasma membrane. This site is based on extraction of iron from the exterior of cells by the impermeable chelator bathophenanthroline disulfonate (BPS). Extraction of iron by BPS inhibits ferricyanide reduction outside the cell, but it is not known if the extraction inhibits oxidase activity [28]. An additional enzyme in plasma membrane which can act as an NADH dehydrogenase is the VDAC pore complex. This enzyme catalyzed NADH ferricyanide reductase activity, but it is not known if it can act as an oxidase or contribute to NADH oxidase activity [25].

## 3. Evidence for Diferric Transferrin As an Electron Acceptor

The stimulation of the plasma membrane NADH oxidase activity by diferric transferrin opens the possibility for a more efficient terminal link to oxygen. There is good evidence that the Fe_2_Tf can act as a terminal oxidase with liver plasma membrane. Two methods have been used to show that diferric transferrin can be reduced by NADH at the plasma membrane [23]. One is by direct measure of the decrease in absorbance of the diferric transferrin at 465 nm when diferric transferrin is in the presence of NADH and isolated plasma membrane. The reduction occurs only in absence of air, and the diferric transferrin absorbance returns immediately when air is admitted [29]. This shows that NADH and plasma membrane can reduce the iron in diferric transferrin anaerobically and that the iron is rapidly reoxidized in the presence of oxygen [23]. The absorbance at 465 nm is characteristic of diferric transferrin [30]. The second assay for reduction of diferric transferrin by NADH and plasma membrane measures the formation of ferrous bathophenanthroline disulfonate (BPS) at 535 nm [23]. This procedure has also been used to show diferric transferrin reduction at the surface of several different types of cells [31–34]. The use of BPS to show the reduction of iron in differic transferrin has been objected to on the basis that BPS effectively raises the redox potential of the ferric transferrin so that it can be reduced by NADH [35]. This is true since the BPS would stabilize the ferrous iron and prevent its reoxidation to ferric by rebinding to the transferrin. The BPS effect would be similar to keeping the iron under anaerobic conditions. The assay does show that electrons from NADH can cross the plasma membrane to reduce the iron in diferric transferrin. Under aerobic conditions without the chelator the ferrous iron would be immediately reoxidized by oxygen to recombine with transferrin and therefore would not be available for transport into the cell as ferrous iron. Thorstensen and Romslo [36] have made an extensive analysis of the relation of the redox system and iron uptake and conclude that at least for hepatocytes the redox system appears to contribute to iron uptake. They point out that anaerobic conditions favor iron uptake by liver cells which is consistent with the effect of argon on the rate of formation of ferrous BPS by liver or HeLa cells incubated with diferric transferrin [37, 38]. Argon increases the rate of ferrous BPS formation by 59% with HeLa cells and by 25% with liver cells. The reduction of ferric ammonium citrate (FAC) is not stimulated under argon [38] which is consistent with much slower reoxidation of ferrous iron not associated with transferrin. The assay of NADH oxidation in the presence of diferric transferrin and plasma membrane provides a corollary to the direct assays of diferric transferrin reduction. The rate of NADH oxidation is increased by addition of diferric transferrin to the plasma membrane. This is consistent with a rapid transfer of electrons to oxygen through diferric transferrin reduction and reoxidation. The stimulation is quite specific for NADH oxidation. With rat liver plasma membranes, diferric transferrin stimulated NADH oxidase 60% and NADPH oxidase 14% [15, 23]. The stimulation was inhibited by the GB16 antibody to the transferrin receptor [39], but increased transferrin receptor in tumor cells does not necessarily increase stimulation [40] which indicates that the transferrin receptor itself is not alone sufficient to act as an oxidase. Diferric lactoferrin is not involved in iron uptake, but is effective in stimulation of cell growth [41, 42]. Diferric lactoferrin also stimulates rat liver plasma membrane NADH oxidase. In a preparation where diferric transferrin stimulated NADH oxidase 73% differic lactoferrin stimulated 80%. For both, the stimulation is inhibited by the transferrin or lactoferrin antibody [33], respectively. Assay with isolated plasma membrane does not show orientation of the enzyme, so the oxidation of internal NADH was measured using whole cells.

## 4. Evidence for a Transmembrane Oxidase

The transmembrane nature of the transferrin-stimulated oxidase can be demonstrated by measuring the cellular concentration of NADH. The oxidation of NAD(P)H in intact K562 cells is seen in the decrease of absorbance at 340 nm when diferric transferrin is added to the cells [34]. This shows that the reduced pyridine nucleotides are oxidized. By using a specific enzymatic assay for NADH, the decrease in HeLa cells on addition of diferric transferrin has been shown [43, 44]. The evidence that addition of diferric transferrin to cells stimulates the immediate oxidation of internal NADH indicating that reduction and reoxidation of differic transferrin on the outside of cells can catalyse oxidation of internal NADH. Inhibition of the transferrin stimulated NADH oxidase by inhibitors of the coenzyme Q pathway is further evidence that the coenzyme Q requiring NADH oxidase is the major site of transferrin stimulation. Capsaicin inhibits diferric transferrin reductase 88% [21], whereas the transmembrane electron transport in neutrophils is not inhibited by the capsaicin analog resiniferatoxin [45]. The coenzyme Q analog piericidin also inhibits rat liver plasma membrane transferrin stimulated NADH oxidase, and the inhibition is partially reversed by coenzyme Q [46].

## 5. The Redox System Involved in Transferrin Reduction

**Figure 1:**
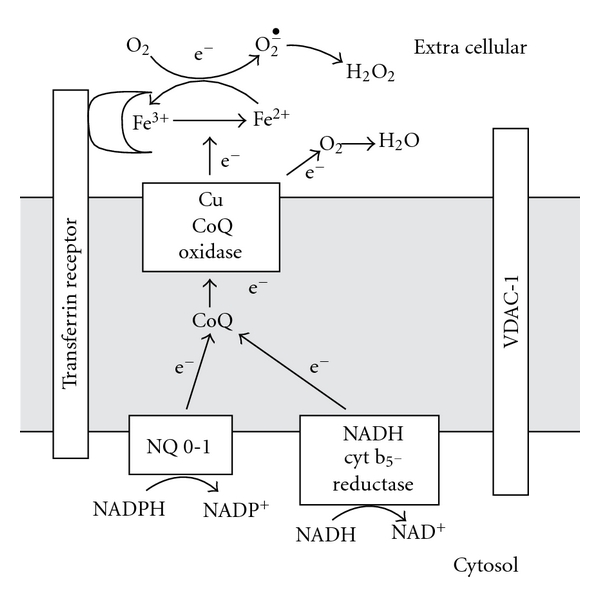
Diagram of plasma membrane redox involved in ferric transferrin reduction [15, 37, 48].

With liver plasma membrane, addition of Fe_2_Tf increases oxidation of both NADH and NADPH. The increase is greater during oxidation of NADH compared to NADPH [15, 23]. There is evidence that oxygen is the terminal acceptor since there is no net reduction of ferric transferrin under aerobic conditions. NADPH has not been tested as an electron donor under anaerobic conditions. Also unknown is which of the transplasma membrane oxidase systems is responsible for the transferrin stimulated oxidation. With liver plasma membrane the dehydrogenase involved is apparently one of the enzymes which requires coenzyme Q as shown by reversible decrease in activity after extraction of CoQ. This would involve either the NADH cytochrome b_5_ reductase or the NADH/NADPH DT diaphorase [26]. There is a report that VDAC-1 can also act as a NADH CoQ reductase so that it is not excluded from a role in transferrin stimulated oxidase [25]. Since there is no evidence that the NADPH oxidase of the NOX1-5 series requires coenzyme Q it is unlikely that they function as the primary transferrin reductase in liver membranes. However it is possible that they transfer electrons through the low-potential cyt b_558_ to diferric transferrin. Also, it should be noted that not all of the Fe_2_Tf-stimulated oxidase was lost after CoQ extraction. The residual activity could be based on NOX-3 which has been identified in liver [47]. The components of the transferrin stimulated NADH oxidase are shown in Figure 1.

## 6. Adventitious Iron

In studies before 1988, we found considerable variation in the rate of diferric transferrin reduction with different preparations of diferric transferrin. Measurement of adventious loosely bound iron using ascorbate as a reductant and BPS to measure formation of ferrous BPS showed that the most active preparations contained up to 1 u mole of loosely bound iron [37] per u mole transferrin. After 1988, diferric transferrin was checked for adventious iron [31, 33, 37]. All the assay procedures done after 1988 with diferric transferrin tested for adventious iron show significant reduction of the diferric transferrin or stimulation of NADH oxidase although the rates are clearly higher if extra iron is present. This is consistent with the stimulation of reduction when ferric ammonium citrate is added along with transferrin as shown in Table 2 [31]. An involvement of the transferrin receptor in activation of the iron reduction may be indicated since the rate with transferrin present can increase over the rate with the same amount of iron [31].

The direct spectrophotometric assay of diferric transferrin reduction by NADH with liver plasma membrane would not be influenced by adventious iron [23]. The inhibition of diferric transferrin reduction by apotransferrin may also be attributed partly to binding of adventious iron to the apotransferrin, since apotransferrin does not always bind strongly to the transferrin receptor [54]. The stimulation of plasma membrane NADH oxidation by different preparations of diferric transferrin is shown in Table 1. The amount of adventious iron was not measured before 1988 so the higher rates probably reflect the presence of excess iron. After 1988, the diferric transferrin was tested for extra iron by the ascorbate reduction test.

These preparations show a more consistent low rate of stimulation. The effect of NAD(P)H oxidase stimulation by diferric transferrin (without extra iron) can be demonstrated by direct spectrophotometric measurement of decrease of NAD(P)H in K562 cells at 340 nm when diferric transferrin is added to the cells [34].

## 7. Function of the Oxidase

The overall effects of diferric transferrin stimulation of the plasma membrane NADH oxidase may be encountered both inside and outside the cells. Inside the oxidation, stimulation can shift the redox poise of the cell by oxidation of the reduced pyridine nucleotide [34] and increase NAD concentration. Outside, transferrin may introduce an alternative source of superoxide by one-electron transfer from ferrous iron to oxygen. Oxidation of NADH by the endogenous oxidase in absence of transferrin is reported not to generate superoxide [24, 55]. A low level of hydrogen peroxide generation by NADH oxidation with liver plasma membranes has been observed [56], but the rate of H_2_O_2_ production was only 3 percent of the rate of oxygen uptake with NADH. The rate of NADPH oxidation which could be a measure of NOX activity was less than 10 percent of the NADH, oxidase but was associated with 50 percent H_2_O_2_ production. 

Superoxide production from NADH at the cell surface can be stimulated by ultraviolet light [57] or phenazine methosulfate [45]. The effect of diferric transferrin on superoxide production at the surface of cells needs to be determined.

Stimulation of the oxidase or transfer of electrons through transferrin iron may increase surface superoxide production above any production by the endogenous oxidase in absence of diferric transferrin. 

A further important function activated by the diferric transferrin is the release of protons from the cells either in conjunction with electron transport or by stimulation of the Na^+^/H^+^antiport. Part of the stimulated proton release is inhibited by amiloride inhibitors of the antiport, and a small part is not inhibited and is stoichiometric to electron transport [33, 50, 58]. Activation of the antiport increases the internal pH and increases membrane potential [38]. The oxidase also controls the concentration of cytosolic NAD which can activate sirtuin that controls metabolic functions [59, 60].

The transferrin stimulated transplasma membrane NADH oxidase may also be involved in control of reactive oxygen species generated in hyperglycaemia or with excess ethanol. Although there are multiple mechanisms involved in hyperglycaemic damage, one aspect is the generation of reactive oxygen species from glycolytic NADH [61]. The major source of ROS based on high glucose in both adipocytes and endothelial cells is mitochondrial pyruvate as indicated by inhibitors. NADH generated by glycolysis in the cytosol did not contribute which suggests that cytosolic NADH is oxidized by the plasma membrane oxidase without generation of ROS [59]. The presence of copper in the oxidase would be consistent with a complete reduction of oxygen to water [48].

On the other hand, in adipocytes, Wu et al. [11] show that the NADH/NADPH oxidase inhibitor diphenyleneiodonium strongly inhibits glucose-stimulated ROS production which suggests inhibition of the plasma membrane oxidase decreases glucose-stimulated ROS generation. With excess ethanol, cells with alcohol dehydrogenase would produce excess cytosolic NADH which would be available for oxidation by the plasma membrane oxidase. Diferric transferrin would increase the oxidation of cytosolic NADH [43] which would lead to formation of ROS if the ferrous iron is reoxidized by a one-electron transfer to oxygen. Thus, the extent of diferric stimulation of the oxidase may influence the damaging effects of excess ethanol. Cells respond to hypoxia by preventing the prolyl hydroxylase-catalyzed breakdown of the transcription factor HIF-1*α* [49]. The diferric transferrin stimulated NADH oxidase may influence the response to mild hypoxia. Generation of ROS decreases ferrous iron to decrease the activity of prolylhydroxylase to decrease the breakdown of HIF-1*α* which would increase the response to mild hypoxia [53] by preserving HIF-1*α*. Oxidation of cytosolic NADH by the plasma membrane oxidase should decrease mitochondrial generation of cytosolic ROS to allow increased HIF-1*α* breakdown. Inhibition of the plasma membrane oxidase should decrease loss of HIF-1*α*. Influence on the level of HIF-1*α* can be important in vital gene regulation and tumor control [52].

Effects on redox state may be enhanced if extra iron is present in the form of ferric citrate since it is clear that extra iron in addition to the two tightly bound atoms in diferric transferrin can further stimulate the plasma membrane oxidase.

Two questions remain unanswered. (A) Does reoxidation of the reduced iron during rebinding to transferrin produce superoxide? and (B) how do functions activated by the transfer of electrons to transferrin affect cell growth or survival [62] and is transferrin stimulation of cell growth based, at least in part, on stimulation of the plasma membrane NADH oxidase?

## 8. Conclusions

Plasma membrane NADPH oxidases are clearly important to plasma membrane signal transduction. Since the plasma membrane NADH oxidase also responds to growth factors like EGF, it may also have a role in signaling. The diferric transferrin stimulation of the NADH oxidase got lost in the discussion of its possible relation to iron uptake. The relation of this transferrin stimulation to the growth stimulation by transferrin remains to be examined. It is likely to play a unique role since ferric lactoferrin which does not participate in iron uptake has also been effective in growth stimulation and in NADH oxidase stimulation. The ferric transferrin stimulation of the oxidase also decreases cytosolic NADH and stimulates the Na^+^/H^+^ antiport which can relate to signal transduction in addition to possible superoxide generation based on the reoxidation of the transferrin iron. The action of excess iron associated with transferrin in further stimulation of the oxidase may relate to problems of iron overload.

## Figures and Tables

**Table 1:** Stimulation of NADH oxidase of rat liver plasma membrane by diferric transferrin (Fe_2_Tf) with control of adventious iron in the Fe_2_Tf.

Year of publication	Fe_2_Tf Concentration micromolar	Extra Fe	NADH oxidase Stimulation *n* mole min −1 mg protein −1	References
1986	3.4	?	3.0	[31]
1987	3.4	?	2.6 + 0.3	[49]
1987	10	?	7.4 + 0.2	[44]
1987	17	?	17	[42]
1990	3.5	?	5.4	[50]
1990	10	NO	0.77 + 0.07	[51]
1991	3	NO	0.9	[8]
1991	10	NO	0.5	[23]
1991	17	NO	1.1	[52]
1992	3.4	NO	2.0	[26]
1992	17	NO	4.5	[26]
1992	12.5	NO	0.91	[53]

**Table 2:** Effect of diferric transferrin on the rate of iron reduction with ferric ammonium citrate (FAC) by SV403T3 cells. Assay with differic transferrin Fe_2_Tf tested for presence of adventious iron. Data from reference [31].

Micromolar additions	Rate of iron reduction (FeBPS formation) *n* mole mm^−1^ cells × 10^−6^
FAC 7.5	0.4
Fe_2_Tf 28	0.6
FAC + Fe_2_Tf	1.9

## Abbreviations

BPS: Bathophenanthroline disulfonate
CoQ10: Coenzyme Q with a 10 isoprene unit side chain
FAC: Ferric ammonium citrate
Fe_2_Tf: Diferric transferrin
cNOX: NADH oxidase bound tightly to membrane
tNOX: NADH oxidase weakly bound to membrane
EGF: Epidermal growth factor
VDAK: Voltage-dependent anion channel
HIF-1*α*: Hypoxia-induced transcription factor.
